# The PROMIZING trial enrollment algorithm for early identification of patients ready for unassisted breathing

**DOI:** 10.1186/s13054-022-04063-4

**Published:** 2022-06-23

**Authors:** Clement Brault, Jordi Mancebo, Juan-Carlos Suarez Montero, Tracey Bentall, Karen E. A. Burns, Thomas Piraino, François Lellouche, Pierre-Alexandre Bouchard, Emmanuel Charbonney, Guillaume Carteaux, Tommaso Maraffi, Gaëtan Beduneau, Alain Mercat, Yoanna Skrobik, Fei Zuo, Myriam Lafreniere-Roula, Kevin Thorpe, Laurent Brochard, Karen J. Bosma

**Affiliations:** 1 Keenan Research Centre for Biomedical Science, Li Ka Shing Knowledge Institute, 209 Victoria St, Toronto, ON Canada; 2 Interdepartmental Division of Critical Care, University of Toronto, Toronto, Canada; 3 Intensive Care Department, Amiens-Picardie University Hospital, Amiens, France; 4 Intensive Care Department, Hospital Universitari de La Santa Creu I Sant Pau, Barcelona, Spain; 5 London Health Sciences Centre, University of Western Ontario, London, ON Canada; 6 Unity Health Toronto - St. Michael’s Hospital, Toronto, ON Canada; 7 Applied Health Research Institute, St. Michael’s Hospital, Toronto, ON Canada; 8 Département de Médecine Québec, Université Laval, Québec City, QC Canada; 9 Institut Universitaire de Cardiologie Et de Pneumologie de Québec, Québec City, QC Canada; 10 Centre de Recherche du Centre Hospitalier de L’Université de Montréal, Montréal, QC Canada; 11 Centre de Recherche du CIUSSS NIM, Montréal, QC Canada; 12 Medical Intensive Care Department, AP-HP, Henri Mondor University Hospital, Créteil, France; 13 Faculté de Santé, Groupe de Recherche Clinique CARMAS, Université Paris Est-Créteil, 94010 Créteil, France; 14 INSERM U955, Institut Mondor de Recherche Biomédicale, 94010 Créteil, France; 15 Service de Réanimation, Centre Hospitalier Intercommunal de Créteil, 40 Avenue de Verdun, 94000 Créteil, France; 16 UNIROUEN, EA 3830, Medical Intensive Care Unit, Rouen University Hospital, Normandie University, 76000 Rouen, France; 17 Medical Intensive Care Unit, Angers University Hospital, Angers, France; 18 Opus Clinic, Montreal, QC Canada; 19 Applied Health Research Centre, Li Ka Shing Knowledge Institute of St. Michael’s Hospital, Toronto, ON Canada; 20 Dalla Lana School of Public Health, University of Toronto and Applied Health Research Centre, Toronto, ON Canada

**Keywords:** Ventilator weaning, Extubation, Mechanical ventilation, Respiratory mechanics, Critical care

## Abstract

**Background:**

Liberating patients from mechanical ventilation (MV) requires a systematic approach. In the context of a clinical trial, we developed a simple algorithm to identify patients who tolerate assisted ventilation but still require ongoing MV to be randomized. We report on the use of this algorithm to screen potential trial participants for enrollment and subsequent randomization in the Proportional Assist Ventilation for Minimizing the Duration of MV (PROMIZING) study.

**Methods:**

The algorithm included five steps: enrollment criteria, pressure support ventilation (PSV) tolerance trial, weaning criteria, continuous positive airway pressure (CPAP) tolerance trial (0 cmH_2_O during 2 min) and spontaneous breathing trial (SBT): on fraction of inspired oxygen (F_i_O_2_) 40% for 30–120 min. Patients who failed the weaning criteria, CPAP Zero trial, or SBT were randomized. We describe the characteristics of patients who were initially enrolled, but passed all steps in the algorithm and consequently were not randomized.

**Results:**

Among the 374 enrolled patients, 93 (25%) patients passed all five steps. At time of enrollment, most patients were on PSV (87%) with a mean (± standard deviation) F_i_O_2_ of 34 (± 6) %, PSV of 8.7 (± 2.9) cmH_2_O, and positive end-expiratory pressure of 6.1 (± 1.6) cmH_2_O. Minute ventilation was 9.0 (± 3.1) L/min with a respiratory rate of 17.4 (± 4.4) breaths/min. Patients were liberated from MV with a median [interquartile range] delay between initial screening and extubation of 5 [1–49] hours. Only 7 (8%) patients required reintubation.

**Conclusion:**

The trial algorithm permitted identification of 93 (25%) patients who were ready to extubate, while their clinicians predicted a duration of ventilation higher than 24 h**.**

**Supplementary Information:**

The online version contains supplementary material available at 10.1186/s13054-022-04063-4.

## Background

Liberating critically ill patients from invasive mechanical ventilation (MV) at the earliest opportunity is essential to avoid the morbidity and mortality associated with prolonged ventilation [1, 2]. However, the process of discontinuing MV is complex. In the “acute phase” of acute respiratory failure and/or uncontrolled critical illness, patients generally receive full ventilator support (i.e., controlled mode of ventilation) to allow the respiratory muscles to rest. In the subsequent “recovery phase,” patients are able to share in the work of breathing. Once patients have improved from the acute phase and meet general “weaning criteria,” daily screening for readiness to wean from MV marks the beginning of the “weaning phase” and is recommended as the best practice to aid early liberation from MV [1]. Currently, there is no consensus for determining when a patient in the acute phase of illness can move to the recovery phase, or even when the patient is able to share the work of breathing. When clinicians switch from a controlled mode to an assisted mode of ventilation may be considered the first step in suspecting readiness to wean. Several studies have evaluated different approaches to managing patients during the weaning phase over the past few decades [3–7]. Weaning typically incorporates spontaneous breathing trials (SBT) using various techniques (low levels of pressure support ventilation (PSV) or continuous positive airway pressure (CPAP) or T-piece) of 30–120 min duration [8–10]. Finally, patients who pass an SBT move to the last “liberation phase,” and are assessed for extubation (see Additional file 6: Fig. E1).

We created a standardized approach for assessing patient's readiness to move from the "acute phase" to the "recovery phase" and then to the "weaning phase" of MV for identifying potential clinical research participants for a randomized controlled trial (RCT) comparing two spontaneous modes of MV. Our purpose here is to describe how the application of this algorithm led to the identification of patients who, previously unsuspected by their clinical team, were actually ready for extubation.

## Methods

### Study protocol

This is an analysis of the enrollment algorithm of the Proportional Assist Ventilation for Minimizing the Duration of Mechanical Ventilation study (PROMIZING, NCT02447692). The PROMIZING study is an ongoing multi-centre randomized controlled trial comparing PSV and proportional assist ventilation with load-adjustable gain factors (PAV +) to facilitate weaning from MV. Informed consent was obtained from the patient or the substitute decision maker at time of enrollment. This early consent allowed the investigators to conduct further tests to determine eligibility for randomization in the PROMIZING Study and to collect minimal information for the screened and non-randomized patients described here. We report here the effects of the enrollment protocol for patients screened between September 2016 and February 2020. We do not report any outcome of the randomized patients.

**Fig. 1:**
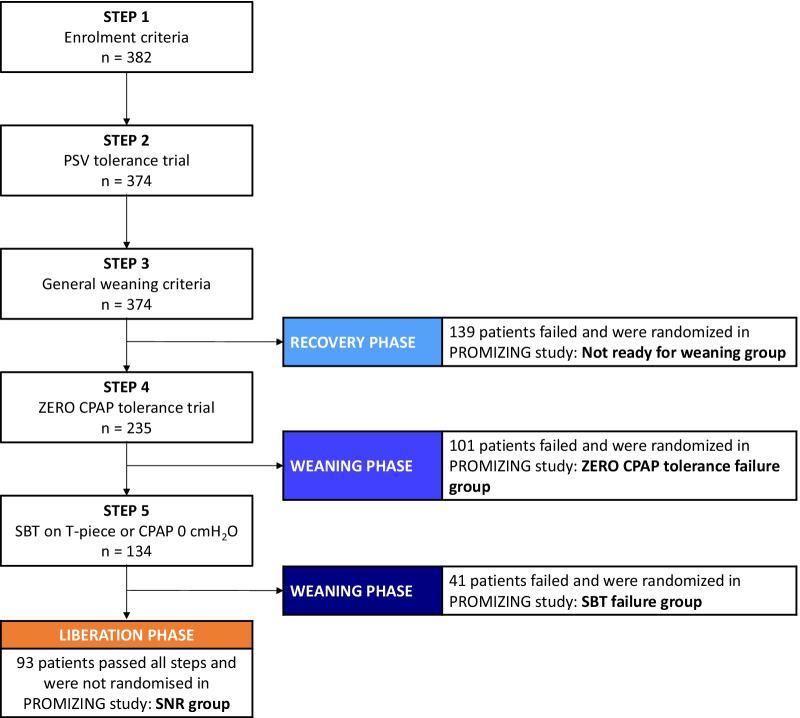
PROMIZING stepwise algorithm and study flowchart. CPAP: continuous positive airway pressure, SNR: screened and non-randomized, PROMIZING: Proportional assist ventilation for minimizing the duration of mechanical ventilation study, PSV: pressure support ventilation, SBT: spontaneous breathing trials

We screened all critically ill patients who received invasive MV for more than 24 h, and who were not expected to be extubated in the next 24 h. Major exclusion criteria were an underlying medical condition likely to result in prolonged or chronic ventilator dependence such as a severe chronic obstructive pulmonary disease or a progressive neuromuscular disorder (the full lists of inclusion and exclusion criteria are provided in Additional file 1: Table E1). After enrollment, further screening tests were done in a step-by-step algorithm to identify patients who were eligible for randomization in the PROMIZING Study to receive either PSV or PAV + (Fig. 1). The goal of this process was to ensure that patients who proceeded to randomization still required continued MV using objective criteria, to ensure that they would not dilute treatment effect (including shortening duration of MV).

### Step 1—Enrollment criteria

Patients satisfying all screening criteria were followed daily until they met the enrollment criteria (see Additional file 2: Table E2). This step ensured (i) that patients were able to trigger ventilator breaths with a reasonable level of assistance, (ii) did not have severe impairment in gas exchange, and (iii) were not hemodynamically unstable. Patients who met enrollment criteria, or their substitute decision makers, were approached for consent. Upon obtaining consent, patients were considered enrolled in the PROMIZING study and ready to undergo further screening tests to determine eligibility for randomization.

### Step 2—Pressure support ventilation tolerance trial

Patients meeting all enrollment criteria were immediately placed on PSV, if they were not already. The PSV tolerance trial (PSVTT) consisted in a pressure of 5–20 cmH_2_O (the total pressure did not exceed 30 cmH_2_O) for at least 30 min [11]. The positive end-expiratory pressure (PEEP) and the fraction of inspired oxygen (F_i_O_2_) settings were similar to that before the PSVTT. If patients were unable to tolerate the PSV because of respiratory distress or clinical instability (see Additional file 3: Table E3), they were immediately returned to the prior ventilation mode. A new PSVTT was attempted at least once daily until patients passed the trial.

### Step 3 – General weaning criteria

Patients who passed the PSVTT were immediately evaluated for general weaning criteria including (i) a peripheral oxygen saturation (S_p_O_2_) ≥ 90% on F_i_O_2_ ≤ 40% and a PEEP ≤ 8 cmH_2_O, (ii) an arterial pH ≥ 7.32, and (iii) vasopressor requirement ≤ 0.1 µg/kg/min of norepinephrine equivalents. Patients who did not meet these three weaning criteria were randomized in the PROMIZING study (Not ready for weaning group). Patients who met these three criteria proceeded directly to a ZERO CPAP tolerance trial to assess their rapid shallow breathing index (RSBI) and capacity to undergo an SBT.

### Step 4 – Zero CPAP tolerance trial

Patients were monitored during a two minute CPAP tolerance trial on their ventilator using a pressure level of 0 cmH_2_O. We assessed the RSBI (as the ratio of respiratory rate to tidal volume, RR/V_t_). Patients who failed this ZERO CPAP tolerance trial due to either a RR/V_t_ ratio > 100, or respiratory distress or clinical instability (see Additional file 4: Table E4) were randomized into the PROMIZING trial (ZERO CPAP tolerance failure group). Patients with a RR/V_t_ ratio ≤ 100 breaths/min/L and S_p_O_2_ ≥ 90% proceeded directly to a SBT.

### Step 5 – Spontaneous breathing trial on T-piece or CPAP 0

Patients were monitored during a 30–120 min SBT on either T-piece or no assistance on the ventilator using CPAP with a pressure level of 0 cmH_2_O as previously described, with F_i_O_2_ 40% [5, 12]. Failure criteria included respiratory distress and clinical instability as for the PSVTT and the ZERO CPAP tolerance trial (see Additional file 4: Table E4). Patients were randomized into the PROMIZING trial if they failed the SBT (SBT failure group). Conversely, patients who passed the SBT were screened and non-randomized (SNR) because they were considered ready for extubation (SNR group).

### Statistical analysis

Demographic, prognostic scores and respiratory parameters were collected before randomization. Continuous variables were expressed as the mean (± standard deviation, SD), or median [interquartile range, IQR]. Categorical variables were quoted as the frequency (percentage). For comparing difference in groups’ characteristics, we used Chi-square test or a one-way analysis of variance (ANOVA) followed by a Tukey’s multiple comparisons post-test when appropriate. All statistical analyses were conducted using R Core Team software (version 4.1.1, R Foundation for Statistical Computing, Vienna, Austria. URL https://www.R-project.org/). Unadjusted p-values are provided to quantify the statistical evidence against equality among the groups in various baseline variables.

## Results

### Study population

Between September 2016 and February 2020, we enrolled (before randomization) 382 patients in the PROMIZING study. Eight patients could not be classified into one of the four groups as they did not complete the pre-randomization assessment or did not have complete data. The mean (± SD) age was 63 (± 13) years and 65% were male. The mean delay between the first intubation and the enrollment was 5.9 (± 5.0) days (Table 1).

**Table 1:** Baseline (pre-randomization) characteristics comparisons

Parameters	All patients n = 374	Not ready for weaning group (Recovery phase) n = 139	ZERO CPAP tolerance failure group (Weaning phase) n = 101	SBT failure Group (Weaning phase) n = 41	SNR Group (Recovery phase) n = 93	*p* value
Age, years	63 ± 13	62 ± 13	63 ± 14	63 ± 13	65 ± 13	0.326
Male – n (%)	242 (65)	95 (69)	58 (59)	30 (75)	59 (65)	0.212
BMI, Kg/m^2^ – median [IQR]	27.8 [24.2–32.1]	28.9 [25.1–34.5]	27.0 [23.5–30.2]	25.9 [23.3–31.2]	28.1 [24.9–32.0]	0.010
RASS	−1.37 ± 1.65	−1.59 ± 1.78	−1.44 ± 1.67	−1.22 ± 1.60	−1.01 ± 1.38	0.062
Postoperative – n (%)	74 (20)	17 (12)	19 (19)	7 (17)	31 (33)	0.001
*Prognostic scores*						
Day 0 SOFA score	6.50 ± 3.35	6.60 ± 3.19	5.73 ± 3.06	5.42 ± 2.70	7.55 ± 3.80	< 0.001^†§^
Charlson Comorbidity Index	4.41 ± 3.13	4.11 ± 3.16	4.31 ± 2.87	5.24 ± 3.74	4.64 ± 3.02	0.216
APACHE III score	77 ± 30	83 ± 27	74 ± 28	71 ± 30	73 ± 35	0.023*
*Mode of ventilation at enrollment*						
Pressure ACV – n (%)	19 (5)	10 (7)	3 (3)	0 (0)	6 (7)	0.189
Volume ACV – n (%)	22 (6)	9 (7)	8 (8)	0 (0)	5 (5)	0.327
PSV – n (%)	325 (87)	118 (85)	88 (85)	40 (98)	79 (85)	0.179
Other (including PAV +) – n (%)	10 (3)	2 (1)	4 (4)	1 (2)	3 (3)	0.665
First intubation to enrollment, days	5.9 ± 5.0	6.6 ± 4.8	6.8 ± 5.9	5.2 ± 4.6	4.5 ± 4.1	NA
*Gas exchange*						
P_a_O_2_/F_i_O_2_	236 ± 81	218 ± 103	260 ± 101	242 ± 70	286 ± 102	< 0.001*
P_a_CO_2_	41.2 ± 8.0	42.3 ± 8.1	41.1 ± 8.9	40.4 ± 6.7	39.8 ± 7.1	0.132
*Respiratory parameters*						
F_i_O_2_, %	38 ± 8	43 ± 9	36 ± 6	37 ± 6	34 ± 6	< 0.001*
Pressure support, cmH_2_O	10.3 ± 3.4	11.1 ± 3.5	10.9 ± 3.2	9.7 ± 3.0	8.7 ± 2.9	< 0.001^*†^
PEEP, cmH_2_O	7.8 ± 2.5	9.7 ± 2.3	7.0 ± 1.7	6.6 ± 1.4	6.1 ± 1.6	< 0.001*^†^
V_t_, mL	516 ± 155	547 ± 171	461 ± 130	514 ± 128	528 ± 151	< 0.001^†^
RR, breaths/min	20.9 ± 6.8	21.7 ± 7.2	23.1 ± 7.3	21.0 ± 6.1	17.4 ± 4.4	< 0.001*^†§^
V_E_, L/min	10.3 ± 3.3	11.2 ± 3.3	10.1 ± 3.2	10.4 ± 3.2	9.0 ± 3.1	< 0.001*

Data presented as mean ± standard deviation unless otherwise stated

Pairwise comparisons between groups by Tukey Honest Significant Difference Test where *p* = 0.05 was taken as a threshold for these post-hoc comparisons:

ACV: assist control ventilation, APACHE: acute physiology and chronic health evaluation, BMI: body mass index, CPAP: continuous positive airway pressure, SNR: screened and non-randomized, F_i_O_2_: fraction of inspired oxygen, IQR: interquartile range, MV: mechanical ventilation, NA: not available, PAV + : proportional assist ventilation, PEEP: positive end-expiratory pressure, PSV: pressure support ventilation, RASS: Richmond agitation and sedation scale, RR: respiratory rate, SBT: spontaneous breathing trial, SOFA: sequential organ failure assessment, V_E_: minute ventilation, V_t_: tidal volume

*Difference (*p* < 0.05) between Not ready for weaning group *vs.* SNR group

^†^Difference (*p* < 0.05) between ZERO CPAP tolerance failure group vs. SNR group

^§^Difference (*p* < 0.05) between SBT failure group vs. SNR group

### Effect of the study algorithm on the mechanical ventilation process

Of the 374 patients, 139 (37%) were randomized because they did not meet general weaning criteria (Not ready for weaning group). Of the remaining 235 patients, 142 patients moved to the next steps: 101 (27%) met general weaning criteria but failed the ZERO CPAP tolerance trial (ZERO CPAP tolerance failure group subsequently randomized); 41 (11%) passed the 2-min CPAP but failed the SBT (SBT failure group subsequently randomized) and 93 (25%) initially screened patients were not randomized in the PROMIZING study, because they were considered already ready for the liberation phase (SNR group) (Fig. 1).

### Distribution of patients in the mechanical ventilation process according to the mode of ventilation

At enrollment, 41 (11%) of the 374 patients analyzed were under assist-control ventilation (ACV). Among them, 19 (46%) progressed to the recovery phase (Not ready for weaning group), 11 (27%) progressed to the weaning phase (ZERO CPAP tolerance and SBT failure groups), and 11 (27%) progressed to the liberation phase (SNR group). Compared with patients with PSV at enrollment (325, 87%), there was no significant difference concerning the percentage of patients in the recovery (118, 36%), weaning (128, 40%) or liberation phase (79, 24%) (p = 0.211, p = 0.119, and p = 0.724; respectively) (Table 2).

**Table 2:** Distribution of patients in the mechanical ventilation process according to the mode of ventilation at enrollment

Study algorithm Group	Mechanical ventilation phase	Patients with ACV n (%)	Patients with PSV n (%)	*p* value
Not ready for weaning group	Recovery	19 (46)	118 (36)	0.211
ZERO CPAP tolerance and SBT failure groups	Weaning	11 (27)	128 (40)	0.119
SNR Group	Liberation	11 (27)	79 (24)	0.724

ACV: assist control ventilation, CPAP: continuous positive airway pressure, PSV: pressure support ventilation, SBT: spontaneous breathing trial, SNR: screened and non-randomized

### Description of screened non-randomized patients

Of the 93 patients included in the SNR group, 59 (65%) were male and the mean (± SD) age was 65 (± 13) years. The mean duration from intubation to enrollment was 4.5 (± 4.1) days. The mean Richmond agitation-sedation scale (RASS) was -1.01 (± 1.38) consistent with drowsy or light sedation (Table 1).

At the time of enrollment, a large majority of patients were under PSV (85%) with a mean F_i_O_2_ of 34 (± 6) %, pressure support of 8.7 (± 2.9) cmH_2_0, and PEEP of 6.1 (± 1.6) cmH_2_O. The mean minute ventilation (V_E_) was 9.0 (± 3.1) L/min with a RR of 17.4 (± 4.4) breaths/min (Table 1).

All patients in the SNR group were extubated with a median [IQR] duration between initial screening and extubation of 5 [1–49] hours, and only 7 (8%) patients required reintubation. Among the latter, two patients subsequently died in intensive care unit (ICU). An additional five patients died in ICU despite getting extubated.

### Comparison of baseline characteristics between non-randomized and randomized patients

Compared to the other groups, the SNR group contained a higher proportion of postoperative patients and had a higher P_a_O_2_/F_i_O_2_ ratio. The time from intubation to enrollment was shorter in the SNR group. Conversely, there was not strong evidence of differences among the groups in age, sex, and RASS. The sequential organ failure assessment (SOFA) on ICU admission was significantly higher in the SNR group, reflecting higher organ dysfunction, but this was not confirmed with other prognostic scores (Table 1).

**Fig. 2:**
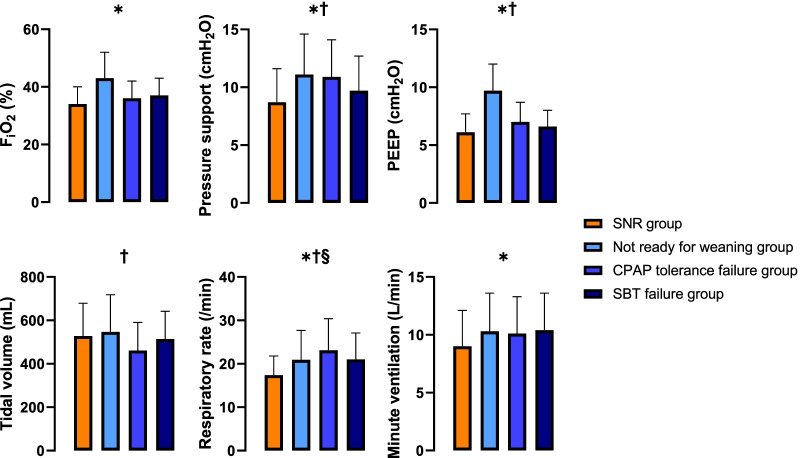
Ventilator settings and respiratory parameters at baseline (pre-randomization) according groups. Data presented as mean ± standard deviation. Pairwise comparisons between groups by Tukey Honest Significant Difference Test where p = 0.05 was taken as a threshold for these post-hoc comparisons: * Difference (p < 0.05) between Not ready for weaning group *vs.* SNR group. † Difference (p < 0.05) between ZERO CPAP tolerance failure group vs. SNR group. § Difference (p < 0.05) between SBT failure group vs. SNR group. SNR: screened and non-randomized, F_i_O_2_: fraction of inspired oxygen, CPAP: continuous positive airway pressure, PEEP: positive end-expiratory pressure, SBT: spontaneous breathing trial

Although the ventilation mode was similar between groups (see Additional file 5: Table E5), the initial ventilator settings slightly differed with a lower level of F_i_O_2_, pressure support and PEEP in the SNR group. These differences were significant when compared with the Not ready for weaning and ZERO CPAP tolerance failure groups, while they disappeared when compared with the SBT failure group. Likewise, the RR was lower in the SNR group compared to the Not ready for weaning group, leading to a significant decrease in V_E_ (Table 1 and Fig. 2).

## Discussion

The major findings of this study are as follow: first, the stepwise algorithm developed for screening and randomizing patients in the PROMIZING trial was helpful in identifying patients who were ready to be separated from the ventilator. Second, a surprisingly large proportion of patients (25% in the present study) for whom clinicians predicted a duration of ventilation higher than 24 h were ultimately determined by the algorithm to be ready for liberation from MV. These patients were extubated within a median delay of 5 h from time of enrollment, and only 7 (8%) required reintubation. This low reintubation rate confirmed that the algorithm was safe [13]. Compared to patients not ready for extubation, they had a slightly lower PSV, PEEP and V_E_. In contrast, the mode of ventilation at enrollment and the level of sedation were similar.

It is difficult for clinicians to determine when a patient is ready to advance from the acute phase of critical illness (requiring full ventilatory support) to the recovery phase (partial ventilatory support) and ultimately, the weaning phase. Several factors could explain this observation such as the absence of simple bedside parameters (e.g., P_a_O_2_/F_i_O_2_ ratio or respiratory system compliance) to predict with certainty the safety and tolerability of allowing patients to share the work of breathing and subsequently start SBTs. Furthermore, the risk of post-extubation respiratory failure requiring reintubation (occurring in up to 15% of cases) is associated with a high mortality rate [13, 14]. Consequently, clinicians frequently tend to underestimate the capacity of patients to be successfully weaned and breathe without assistance, leading to a risk of delayed extubation and exposing patients to unnecessary discomfort and complications (e.g., ventilation-acquired pneumonia) [1]. To avoid delays in the weaning process, international guidelines suggested the implementation of a ventilator liberation protocol to identify patients ready for extubation [3, 15, 16]. To date, guidelines have focused on the weaning phase of MV by proposing a daily interruption of sedation and performing a SBT (PSV with low support pressure, CPAP or T-piece trial) as soon as possible [17]. Because the absence of clear guidance concerning the recovery phase (i.e., when to switch the ventilator from a controlled mode to an assisted mode, and perform these tests) and a lack of objective criteria for deciding if a patient is ready for extubation, our algorithm might add substantial improvements to the current recommendations.

We developed a step-by-step algorithm for patient recruitment in the PROMIZING trial, which could accelerate the weaning from MV. By different ways, it allowed to easily select patients potentially ready to be extubated. First, our algorithm screened early in the MV process, from the acute phase of their illness, when some patients were still in ACV. We proposed simple criteria to switch from ACV to PSV such as adequate gas exchange (with F_i_O_2_ ≤ 60% and PEEP ≤ 15 cmH_2_O) and no hemodynamic instability. Thus, we encouraged a PSVTT as soon as the patient’s condition shows early signs of improvement, but even before patients were seen to trigger the ventilator or before sedatives were weaned or vasopressors were discontinued. Among our 41 patients with ACV at enrollment, our algorithm detected that 19 patients were ready to progress to the recovery (i.e., switch on PSV) phase, and 11 to the weaning phase (i.e., SBT). Moreover, 11 patients successively passed all phases of our algorithm and were ready for extubation. Of note, patients with ACV and PSV at enrollment had a similar extubation rate (27 *vs.* 24%; p = 0.724). This strategy to transfer the breathing workload as soon as possible might limit the development of diaphragm weakness and atrophy, which can occur during the first days of MV [18]. Several studies found a relationship between the diaphragm thickness and weaning failure, delays in liberation of MV or risk of reintubation [19–22].

Second, our protocol might shorten the length of MV, because we used pragmatic criteria to progress from the recovery phase to the weaning phase. Currently, the list of weaning criteria proposed by Boles et al. includes clinical assessment (e.g., adequate cough and no excessive tracheobronchial secretion) and hemodynamic and respiratory measurements [1]. However, some patients who do not meet all the criteria could successfully pass the SBT. In contrast, we moved patients to the weaning phase if they met only three simple criteria: adequate oxygenation (S_p_O_2_ ≥ 90% on F_i_O_2_ ≤ 40% and PEEP ≤ 8 cmH_2_O), no severe acidosis (pH ≥ 7.32), and low-dose of vasopressors. Although the discontinuation of vasopressors has been a precondition for SBT in clinical trials and guidelines, other studies have not found significant differences between patients extubated on and off vasopressors concerning the success of extubation, at least with low doses [23, 24]. In addition, previous studies have shown that patients extubated on vasopressors had a significant decrease in the ICU length of stay [24, 25]. Finally, we conducted the SBT regardless of the PSV or PEEP level (i.e., a gradual withdrawal of assistance was not necessary as long as the pressure support was between 5 and 20 cmH_2_O). Thereby, the duration of the recovery phase could be very short in some of our patients, because they met the general weaning criteria immediately after passing the 30 min PSVTT.

Third, our algorithm defined and separated the different phases of the MV process (i.e., acute, recovery, weaning and liberation phases), and proposed criteria for identifying patients in each phase (see Additional file 6: Fig. E1). In this way, the diagnosis of a possible liberation from MV could be assessed over a few hours. Currently, there is no consensus in the definition of the different phases, often leading to confusion [26]. Using our algorithm, the acute phase corresponded to a non-controlled underlying disease, when patients may need full ventilator support (i.e., ACV). The recovery phase began with the switch to PSV (or proportional ventilation mode), allowing the patient to share the work of breathing. Moving to the weaning phase required meeting the general weaning criteria including a 2-min CPAP trial to assess the RSBI (conducted on zero PEEP) followed by a SBT if the ZERO CPAP tolerance trial was successful. Recently, Burns et al. showed a large heterogeneity of SBT techniques across the world [8]. Our protocol proposed to perform an 30–120 min SBT on T-piece or CPAP at zero PEEP on the ventilator rather than a PSV using low level of pressure support in order to better simulate the physiologic conditions after extubation [5]. Because usual criteria of successful SBT were subjective and depended on the clinician’s interpretation, we provided easy to use criteria to define failure of each PSV, CPAP and T-piece trials [27].

Several limitations of this study need to be discussed. We did not record data regarding the cumulative dose and duration of sedative drugs received or the patient’s fluid balance, which may influence the weaning process [28, 29]. Moreover, our algorithm did not include additional physiologic measurements, such as the airway occlusion pressure (P_0.1_). Finally, in spite of broad and easy-to-assess enrollment criteria (especially concerning oxygenation: P_a_O_2_ ≥ 60 mmHg on F_i_O_2_ ≤ 60% and PEEP ≤ 15 cmH_2_O), the delay between intubation and the enrollment remained long (around 6 days). This finding may also represent an opportunity for improvement as patients could be enrolled earlier in the acute phase. Because a criterion to enroll patients in PROMIZING was an expected need of ventilation of at least 24 h, it is unlikely that clinicians would have conducted a weaning trial independent from the study screening protocol on the same day, suggesting that application of the screening algorithm assisted in identifying patients ready for weaning earlier than the clinicians suspected.

## Conclusion

The process to liberate patients from MV accounts for a significant duration of the ventilation time. Finding strategies to minimize this duration is desirable. We developed a comprehensive step-by-step algorithm for enrollment and randomization of patients in the PROMIZING study, which compares two spontaneous modes of MV. Surprisingly, our algorithm allowed an easy and early identification of patients ready to extubate, and might decrease the duration of MV. In our study, 25% of our patients, who were in need of ventilator assistance for at least 24 h according to their clinicians in charge, passed the whole process (recovery, weaning, and liberation) and were safely removed from the ventilator and extubated.

## Supplementary Information

**Additional file 1** Screening inclusion and exclusion criteria of the PROMIZING study. FEV1: forced expiratory volume in the first second, GOLD: global initiative for chronic obstructive lung disease, ICU: intensive care unit, MRC: medical research council, pCO2: partial pressure of carbon dioxyde, PROMIZING: Proportional assist ventilation for minimizing the duration of mechanical ventilation study.

**Additional file 2** Enrollment inclusion, deferral and exclusion criteria of the PROMIZING study.CPAP: continuous positive airway pressure, ECMO: extracorporeal membrane oxygenation,PaO2: Arterial partial pressure of oxygen PAV: proportional assist ventilation, PEEP: positive end-expiratory pressure, PROMIZING: Proportional assist ventilation for minimizing the duration of mechanical ventilation study, SpO2: peripheral oxygen saturation.

**Additional file 3** Pressure support ventilation tolerance trial inclusion, deferral and exclusion criteria of the PROMIZING study.PROMIZING: Proportional assist ventilation for minimizing the duration of mechanical ventilation study.

**Additional file 4** Definition of respiratory distress and clinical instability.RASS: Richmond agitation sedation scale, SBP: systolic blood pressure, SpO2: peripheral oxygen saturation.

**Additional file 5** Mode of ventilation at baseline (pre-randomization).CPAP: continuous positive airway pressure, SBT: spontaneous breathing trial, SNR: screened and non-randomized.

**Additional file 6** Principles and objectives of each phases of the mechanical ventilation process in the PROMIZING study.Step 1 to 5 refer to the algorithm for enrollment of patients in the PROMIZING study.ACV: assist-control ventilation, CPAP: continuous positive airway pressure, PAV: proportional assist ventilation, PROMIZING: Proportional assist ventilation for minimizing the duration of mechanical ventilation study, PSV: pressure support ventilation, SBT: spontaneous breathing trials.

## Data Availability

The raw data supporting the conclusions of this article will be made available by the authors, on reasonable request.

## Abbreviations

ACV: Assist-control ventilation
ANOVA: Analysis of variance
CPAP: Continuous positive airway pressure
SNR: Screened and non-randomized
F_i_O_2_: Fraction of inspired oxygen
ICU: Intensive care unit
IQR: Interquartile range
MV: Mechanical ventilation
P_0.1_: Airway occlusion pressure
P_a_O_2_: Partial pressure of oxygen
PAV +: Proportional assist ventilation with load-adjustable gain factors
PEEP: Positive end-expiratory pressure
PROMIZING: Proportional assist ventilation for minimizing the duration of mechanical ventilation study
PSVTT: Pressure support ventilation tolerance trial
PSV: Pressure support ventilation
RASS: Richmond agitation-sedation scale
RR/V_t_: Ratio of respiratory rate to tidal volume
SBT: Spontaneous breathing trials
SD: Standard deviation
SOFA: Sequential organ failure assessment
S_p_O_2_: Peripheral oxygen saturation
V_E_: Minute ventilation
