# Supplementary material for: The PROMIZING trial enrollment algorithm for early identification of patients ready for unassisted breathing
Source: Crit Care. 2022 Jun 23;26:188. doi: 10.1186/s13054-022-04063-4 (PMC9219177; doi:10.1186/s13054-022-04063-4)
Supplement: Supplementary file 1 — Additional file 1 Screening inclusion and exclusion criteria of the PROMIZING study. FEV1: forced expiratory volume in the first second, GOLD: global initiative for chronic obstructive lung disease, ICU: intensive care unit, MRC: medical research council, pCO2: partial pressure of carbon dioxyde, PROMIZING: Proportional assist ventilation for minimizing the duration of mechanical ventilation study. [file 13054_2022_4063_MOESM1_ESM.docx]

| **Screening phase** | | |
| --- | --- | --- |
|  | **Inclusion criteria** | |
|  | 1. | Age ≥ 18 years |
|  | 2. | Intubated and receiving any mode of invasive mechanical ventilation ≥ 24 hours |
|  | **Exclusion criteria** | |
|  | 1. | Anticipating withdrawal of life support and/or shift to palliation as the goal of care |
|  | 2. | Severe central neurologic disorder (e.g., haemorrhage, stroke, tumour) causing elevated intracranial pressure, or impaired control of breathing, or requiring specific ventilator adjustments (i.e., to attain specific CO_2_ target) or requiring neurosurgical intervention |
|  | 3. | Known or suspected severe or progressive neuromuscular disorder likely to result in prolonged or chronic ventilator dependence (e.g., Guillain-Barré syndrome, myasthenia gravis, amyotrophic lateral sclerosis, multiple sclerosis, high spinal cord injury, kyphoscoliosis, or other restrictive disorder) (Note that obesity hypoventilation syndrome that may be managed with nocturnal non-invasive ventilation is not an exclusion under A5) |
|  | 4. | Severe chronic obstructive pulmonary disease: baseline daytime hypercapnia (pCO_2_ > 50 mmHg) OR GOLD 4 airflow limitation (FEV1 < 30% predicted) OR medical research council (MRC) class 4 symptoms (“I am too breathless to leave the house” OR “I am breathless when dressing”) |
|  | 5. | Broncho-pleural fistula |
|  | 6. | Tracheostomy present at intensive care unit (ICU) admission for the purpose of chronic or prolonged mechanical ventilation (> 21 days) (Note that a patient who was endotracheally intubated for acute respiratory failure and received a tracheostomy during their ICU admission, prior to enrolment, is not excluded under A8) |
|  | 7. | Current enrolment in a confounding study, as assessed by the steering committee |
|  | 8. | Previous randomization in the PROMIZING Study |
|  | 9. | Severe, end-stage, irreversible respiratory or cardiac disease (e.g. interstitial lung disease, pulmonary fibrosis, cardiomyopathy, valvulopathy) likely to result in prolonged or chronic ventilator dependence /unlikely to wean from mechanical ventilation (Note that patients who are candidates for intervention to treat the underlying respiratory/cardiac disease (e.g. lung transplant, heart transplant, cardiac surgery) may be re-evaluated once intervention is complete and they no longer meet criteria A11) |
