# Supplementary material for: The PROMIZING trial enrollment algorithm for early identification of patients ready for unassisted breathing
Source: Crit Care. 2022 Jun 23;26:188. doi: 10.1186/s13054-022-04063-4 (PMC9219177; doi:10.1186/s13054-022-04063-4)
Supplement: Supplementary file 2 — Additional file 2 Enrollment inclusion, deferral and exclusion criteria of the PROMIZING study.CPAP: continuous positive airway pressure, ECMO: extracorporeal membrane oxygenation,PaO2: Arterial partial pressure of oxygen PAV: proportional assist ventilation, PEEP: positive end-expiratory pressure, PROMIZING: Proportional assist ventilation for minimizing the duration of mechanical ventilation study, SpO2: peripheral oxygen saturation. [file 13054_2022_4063_MOESM2_ESM.docx]

| **Enrolment phase** | | |
| --- | --- | --- |
|  | **Inclusion criteria** | |
|  | 1. | Ability or potential ability to trigger ventilator breaths (i.e. not receiving neuromuscular blockade) |
|  | 2. | On assist control volume-cycled ventilation: Technically satisfactory plateau pressure ≤ 30 cmH_2_O OR on assist control pressure-controlled ventilation or similar mode: pressure control plus positive end-expiratory pressure (PEEP) ≤ 30 cmH_2_O OR on pressure support ventilation: pressure support plus PEEP ≤ 30 cmH_2_O OR on proportional assist ventilation (PAV): PAV gain < 85% |
|  | 3. | Arterial partial pressure of oxygen (P_a_O_2_) ≥ 60 mmHg or peripheral oxygen saturation (S_p_O_2_) ≥ 90% on F_i_O_2_ ≤ 60% and PEEP ≤ 15 cmH_2_O |
|  | 4. | Metabolic disorders corrected: pH ≥ 7.32 |
|  | 5. | Stable hemodynamic status: stable or decreasing doses of vasopressors for ≥6 hours |
|  | 6. | Anticipate ongoing need for ventilation > 24 hours |
|  | **Deferral criteria** | |
|  | 1. | Plan to extubate/discontinue mechanical ventilation within < 24 hours |
|  | 2. | Patient currently on extracorporeal membrane oxygenation (ECMO) |
|  | 3. | Plan for surgery or complex procedure that will require full ventilation to be done prior to attempting extubation (e.g. procedure requiring neuromuscular blockade and/or heavy sedation, such that patient would be apneic, or not be able to trigger ventilator) |
|  | **Exclusion criteria** | |
|  | 1. | Extubated |
|  | 2. | Died |
|  | 3. | Patient has met enrolment inclusion criteria B1-B5 AND has tolerated pressure support of 0-20 cmH_2_O or PAV gain 0-85% for ≥ 24 consecutive hours (including time on continuous positive airway pressure (CPAP), T-piece, or tracheostomy mask)  Note (1): that it is acceptable to include a patient who has been tried on pressure support or proportional assist ventilation but has required pressures > 20 cmH_2_O or PAV gain > 85% or has required return to assist control ventilation within the 24-hour time window  Note (2): B9 does not apply to patients on ECMO |
|  | 4. | Patient transferred to a non-participating centre |
