# Supplementary material for: The PROMIZING trial enrollment algorithm for early identification of patients ready for unassisted breathing
Source: Crit Care. 2022 Jun 23;26:188. doi: 10.1186/s13054-022-04063-4 (PMC9219177; doi:10.1186/s13054-022-04063-4)
Supplement: Supplementary file 3 — Additional file 3 Pressure support ventilation tolerance trial inclusion, deferral and exclusion criteria of the PROMIZING study.PROMIZING: Proportional assist ventilation for minimizing the duration of mechanical ventilation study. [file 13054_2022_4063_MOESM3_ESM.docx]

| **Pressure support ventilation tolerance trial (PSVTT)** | | |
| --- | --- | --- |
|  | **Inclusion criteria** | |
|  | 1. | Upon review of Screening and Enrolment criteria (cf. Table S1), the patient still passes |
|  | 2. | Treating physician has provided verbal consent to proceed with standardized tests and randomization if eligibility criteria are met |
|  | **Deferral criteria** | |
|  | 1. | High dose vasopressor requirements (i.e. epinephrine or norepinephrine > 0.5 µg/kg/min or equivalent) OR patient requiring an increase in dose of vasopressor within 6 hours |
|  | 2. | Active cardiac ischemia (dynamic ST changes on monitor or electrocardiogram within 6 hours) |
|  | 3. | Unstable arrhythmias (heart ratio > 140 or < 50 beats/min) with clinical signs of low cardiac output OR systolic blood pressure < 80 mmHg |
|  | 4. | Receiving a “strict lung protective” ventilation strategy for acute respiratory distress syndrome |
|  | **Exclusion criteria** | |
|  | 1. | Treating physician has declined consent |
