# Supplementary material for: The PROMIZING trial enrollment algorithm for early identification of patients ready for unassisted breathing
Source: Crit Care. 2022 Jun 23;26:188. doi: 10.1186/s13054-022-04063-4 (PMC9219177; doi:10.1186/s13054-022-04063-4)
Supplement: Supplementary file 4 — Additional file 4 Definition of respiratory distress and clinical instability.RASS: Richmond agitation sedation scale, SBP: systolic blood pressure, SpO2: peripheral oxygen saturation. [file 13054_2022_4063_MOESM4_ESM.docx]

| **Respiratory distress** | |
| --- | --- |
| At least two of the following: | |
| 1. | Peripheral oxygen saturation (S_p_O_2_) < 90% |
| 2. | Sustained (> 5 min) respiratory rate > 35 breaths/min |
| 3. | Heart rate > 140 beats/min or a sustained (> 5 min) increase of 20 % from baseline |
| 4. | Systolic blood pressure (SBP) > 180 or < 80 mmHg and/or SBP changes > 30% from baseline |
| 5. | Increased anxiety |
| 6. | Use of accessory muscles |
| 7. | Complaint of dyspnea |
| 8. | Diaphoresis |
| **Clinical instability** | |
| Any one of the following: | |
| 1. | Unstable hemodynamic status (SBP < 80 mmHg) with or without vasoactive drug |
| 2. | Vasopressor requirements > 0.5 µg/kg/min epinephrine, norepinephrine or equivalent |
| 3. | Active cardiac ischemia (dynamic ST changes on cardiac monitor or electrocardiogram) |
| 4. | Unstable arrhythmias (heart rate > 140 or < 50 beats/min) with clinical signs of low cardiac output or SBP < 80 mmHg |
| 5. | Uncontrolled hypertension (SBP > 180 mmHg) |
| 6. | Abrupt decrease in the level of consciousness (Richmond agitation sedation scale [RASS] -4 or -5 OR sedation agitation scale [SAS] 1 or 2) |
| 7. | Dangerous agitation (RASS +4 or +3 or SAS 7) |
| 8. | Metabolic (or mixed) acidosis with pH < 7.32 |
| 9. | Emergency situation that merits return to assist control ventilation according to best clinical judgement |
