# Supplementary material for: The PROMIZING trial enrollment algorithm for early identification of patients ready for unassisted breathing
Source: Crit Care. 2022 Jun 23;26:188. doi: 10.1186/s13054-022-04063-4 (PMC9219177; doi:10.1186/s13054-022-04063-4)
Supplement: Supplementary file 5 — Additional file 5 Mode of ventilation at baseline (pre-randomization).CPAP: continuous positive airway pressure, SBT: spontaneous breathing trial, SNR: screened and non-randomized. [file 13054_2022_4063_MOESM5_ESM.docx]

| **Parameters** | **Not ready for weaning group**  **(Recovery phase)**  **n = 139** | **ZERO CPAP tolerance failure group**  **(Weaning phase)**  **n = 101** | **SBT failure**  **Group**  **(Weaning phase)**  **n = 41** | **SNR Group**  **(Liberation phase)**  **n = 93** | ***p* value** |
| --- | --- | --- | --- | --- | --- |
| Assist/Control Ventilation – n (%) | 19 (14) | 11 (11) | 0 (0) | 11 (12) | 0.262 |
| Pressure Support Ventilation – n (%) | 118 (85) | 86 (85) | 40 (98) | 79 (85) |  |
| Other mode of ventilation – n (%) | 2 (1) | 4 (4) | 1 (2) | 3 (3) |  |
