# Supplementary material for: The PROMIZING trial enrollment algorithm for early identification of patients ready for unassisted breathing
Source: Crit Care. 2022 Jun 23;26:188. doi: 10.1186/s13054-022-04063-4 (PMC9219177; doi:10.1186/s13054-022-04063-4)
Supplement: Supplementary file 6 — Additional file 6 Principles and objectives of each phases of the mechanical ventilation process in the PROMIZING study.Step 1 to 5 refer to the algorithm for enrollment of patients in the PROMIZING study.ACV: assist-control ventilation, CPAP: continuous positive airway pressure, PAV: proportional assist ventilation, PROMIZING: Proportional assist ventilation for minimizing the duration of mechanical ventilation study, PSV: pressure support ventilation, SBT: spontaneous breathing trials. [file 13054_2022_4063_MOESM6_ESM.pptx]

## Slide 1
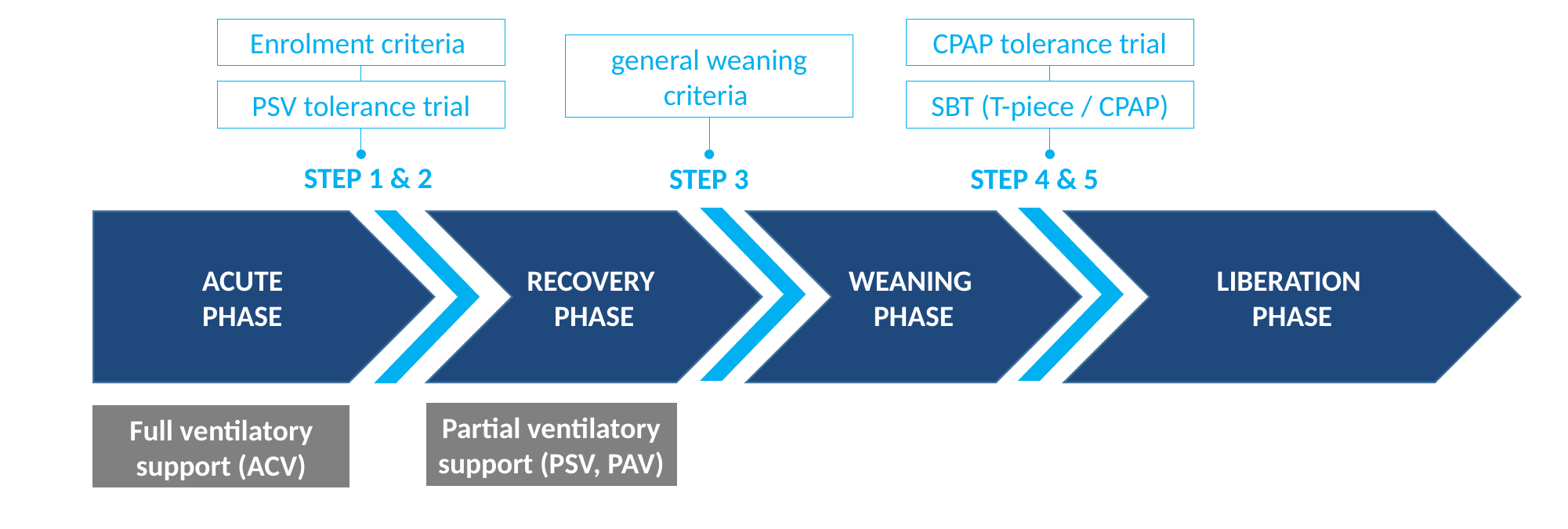

Enrolment criteria
CPAP tolerance trial
general weaning criteria
PSV tolerance trial
SBT (T-piece / CPAP)
STEP 1 & 2
STEP 3
STEP 4 & 5
ACUTE
PHASE
RECOVERY
PHASE
WEANING
PHASE
LIBERATION
PHASE
Partial ventilatory support (PSV, PAV)
Full ventilatory support (ACV)
